# Intrinsic Motivation, Attitudes, and Practices of Young Physicians Regarding Scientific Research: Cross-Sectional Study in China

**DOI:** 10.2196/72633

**Published:** 2025-04-22

**Authors:** Liwei Wang, Jiajia Ren, Song Zhang, Yu’e Sun, Yu Ding, Congxian Yang, Chan Zheng, Zhenduo Shi, Yangzi Zhu

**Affiliations:** 1 Department of Anesthesiology Xuzhou Clinical School of Xuzhou Medical University Xuzhou Central Hospital Xuzhou China; 2 Department of Science and Education Xuzhou Clinical School of Xuzhou Medical University Xuzhou Central Hospital Xuzhou China; 3 Department of Anesthesiology Renji Hospital and Shanghai Jiaotong University School of Medicine Shanghai China; 4 Department of Anesthesiology Nanjing Drum Tower Hospital Nanjing University Nanjing China; 5 Department of Neurosurgery The Affiliated Suqian Hospital of Xuzhou Medical University Suqian China; 6 Department of Pain Management Shandong Provincial Hospital Affiliated to the Shandong First Medical University Jinan China; 7 Department of Pain Management First Affiliated Hospital of Xiamen University Xiamen China; 8 Department of Urology Xuzhou Central Hospital Xuzhou China; 9 Jiangsu Province Key Laboratory of Anesthesiology Xuzhou Medical University Xuzhou China

**Keywords:** intrinsic motivation, attitude, practice, scientific research, young physicians, cross-sectional study

## Abstract

**Background:**

Recent decades have witnessed a concerning global trend of declining engagement among physician scientists, with participation rates falling from 4.7% in the 1980s to approximately 1.5% today in the United States. The research highlights the declining engagement of physician scientists and the challenges young physicians face in participating in clinical research.

**Objective:**

This study aims to examine the intrinsic motivation, attitudes, and practices of young physicians toward scientific research and its clinical value and identify factors that influence their engagement in research activities.

**Methods:**

We developed a comprehensive questionnaire measuring intrinsic motivation (27 items; score range 27-135), attitudes (8 items; score range 8-40), and practices (7 items; score range 7-35) related to scientific research among physicians. Cronbach α coefficients for the 3 dimensions were 0.967, 0.916, and 0.937, respectively. A cross-sectional survey was conducted on young physicians from 12 hospitals in eastern provinces of China between May 2024 and October 2024.

**Results:**

A total of 532 valid questionnaires were obtained. Among the respondents, 271 (50.9%) were female, and 317 (59.6%) had not led or been deeply involved in a research project. Most physicians (more than 80%) reported high intrinsic motivation and positive attitudes, but relatively fewer demonstrated active research practices. Key challenges identified included balancing research with clinical work (n=102, 19.2%) disagreed that research alleviates clinical monotony) and insufficient institutional support (n=329, 61.3%) reported inadequate research investment from their hospitals). The mean scores for intrinsic motivation, attitude, and practice were 108.79 (SD 11.91; possible range: 27-135), 32.23 (SD 4.27; possible range: 8-40), and 27.44 (SD 3.81; possible range: 7-35), respectively. Multivariate logistic regression showed that intrinsic motivation score (odds ratio [OR] 1.063, 95% CI 1.035-1.091), attitude score (OR 1.095, 95% CI 1.029-1.165), and good research atmosphere (OR 1.915, 95% CI 1.038-3.533) were independently associated with practice. Moreover, structural equation modeling analysis revealed that intrinsic motivation had a direct effect on attitude (β=0.854; *P*<.001), attitude directly affected practice (β=0.637; *P*<.001), and intrinsic motivation indirectly influenced practice through attitude (β=0.544; *P*<.001).

**Conclusions:**

Despite high levels of intrinsic motivation and positive attitudes toward research, young physicians face significant barriers to active research engagement. Our findings suggest that fostering a supportive research environment is a critical factor that can help translate motivation into practice. Young physicians exhibited positive intrinsic motivation and attitudes but relatively inactive practices toward scientific research and its clinical application. Institutional initiatives should focus on providing protected research time, formal mentorship programs, and adequate research infrastructure to leverage young physicians’ existing motivation. Addressing the gap between motivation and practice could significantly contribute to reversing the declining trend of physician scientists and enhancing evidence-based medicine implementation.

## Introduction

Current medical advancements have reached new heights, underscoring the critical role of physician scientists in this process. However, domestic and international studies reveal a concerning trend: the proportion of clinical physicians engaged in scientific research has steadily declined in recent years. This decline represents a critical challenge for the advancement of medical science and highlights the need to understand the factors influencing research engagement. For instance, the percentage of US physicians conducting research dropped from a peak of 4.7% in the 1980s to approximately 1.5% today, as highlighted by Nobel laureates and other prominent medical scientists in a 2019 *The New England Journal of Medicine* paper calling for urgent measures to “save the endangered physician scientist.” The authors characterized physician scientists as an “endangered species” and emphasized that this decline threatens the translation of scientific discoveries into clinical applications. They identified several critical factors contributing to this trend, including increasing clinical demands, financial pressures, lengthening training periods, and insufficient mentorship. Their urgent call for systemic interventions to reverse this decline underscores the gravity of the situation and its potential impact on future medical advances [1]. Similarly, recent studies indicate a growing need to understand the factors influencing research engagement among young physicians and identify strategies to address these challenges [2].

Physicians are expected to conduct research in diverse clinical and educational settings and disseminate their findings to advance medicine and health care. However, even motivated novice researchers face significant psychological and procedural barriers, including uncertainty about research methods, lack of confidence in their abilities, and confusion about where to begin the scientific inquiry process [3]. These barriers are compounded by practical difficulties identified across multiple studies. Raffing et al [3] specifically highlighted the challenges facing young medical doctors in writing their first paper, finding that inadequate training in scientific writing and statistical analysis were significant barriers. Khan et al [4] conducted a comprehensive survey of physicians-in-training in Pakistan, revealing that time constraints and limited funding opportunities were the most frequently cited obstacles to research engagement, with over 80% of respondents identifying these as major barriers. Similarly, Mitwalli et al [5] found in their study of resident physicians in Saudi Arabia that inadequate mentorship and communication challenges for nonnative English speakers significantly impacted research productivity and publication rates. Understanding these barriers is essential, but equally important is gaining insight into the psychological and motivational factors that drive research engagement among physicians.

Despite extensive research on physician scientists, significant gaps exist in the literature. Few studies have focused specifically on young physicians at early career stages, especially in China. The relationship between intrinsic motivation, attitudes, and actual research practices remains underexplored. Additionally, most previous studies lack advanced statistical methods such as structural equation modeling to understand the complex interrelationships among these factors. Our study addresses these gaps by examining these dimensions among young Chinese physicians using a comprehensive analytical approach. Understanding the intrinsic motivation, attitudes, and practices of young physicians toward scientific research is particularly important, as this group is at a pivotal stage in their careers. Research engagement at this stage can shape their long-term contributions to medical advancements and evidence-based practice. Targeting young physicians allows us to identify barriers and enablers to research participation early, enabling the development of tailored interventions to foster a culture of research. By addressing these challenges, the study aims to bridge the gap between research and practice, ensuring young physicians are equipped not only with the skills and motivation to conduct research but also with the capacity to apply their findings to improve health care quality and patient outcomes. To effectively address these challenges, a theoretical framework is needed to understand the complex relationships between motivation, attitudes, and research practices.

The Knowledge, Attitude, and Practice (KAP) theory provides a useful framework for understanding behavior change, emphasizing that knowledge serves as the foundation for change, while attitudes and beliefs act as the driving forces behind behavioral transformation [6]. In designing this study, we referenced KAP theory but intentionally chose to focus on the more dynamic and behavior-driven dimensions of intrinsic motivation, attitudes, and practices. This decision acknowledges the diverse research skills and methodological knowledge required in different medical fields, which make the “knowledge” dimension less universally applicable. Several previous studies support this adaptation, including Johnson et al [7], who found that motivation rather than knowledge was the primary predictor of research engagement among health care professionals. Furthermore, our focus on intrinsic motivation rather than knowledge allows us to examine the psychological drivers that sustain research engagement over time, addressing what Bakken et al [8] identified as the “motivation-action gap” in physician scientist development. Instead, intrinsic motivation, attitudes, and practices are more directly linked to whether physicians actively engage in scientific research and apply it in clinical settings. Given the importance of these factors and the limited research in this area, particularly in the Chinese context, our study aims to fill this critical gap.

To our knowledge, after a systematic search, we found limited research on this topic in China. Existing studies have primarily focused on medical students’ research competencies [9], research output metrics of established researchers [10], or barriers to research in specific specialties [11]. However, there is a lack of comprehensive studies examining the psychological factors and behavioral dimensions of research engagement specifically among young Chinese physicians. Thus, this study aims to examine the intrinsic motivation, attitudes, and practices of young physicians regarding scientific research and its clinical value.

## Methods

### Study Design and Participants

This cross-sectional study was conducted on young physicians from 12 hospitals in the eastern provinces of China (Shandong, Jiangsu, Shanghai, Zhejiang, and Fujian) between May 2024 and October 2024. The inclusion criteria are (1) physicians aged≤45 years and (2) physicians with valid medical practice licenses. The exclusion criteria are physicians who declined to participate in the survey or did not provide informed consent.

### Ethical Considerations

This study received a formal exemption from requiring ethical approval by the Ethics Committee of Xuzhou Central Hospital due to its noninterventional, survey-based design. The informed consent was secured from all participants. All data were anonymized and no compensation was provided to the participants.

### Questionnaire Design

After the initial design of the questionnaire, modifications were made based on feedback from 3 experts. A small-scale pilot distribution involving 49 samples was then conducted to assess its reliability. The results demonstrated high internal consistency, with an overall Cronbach α coefficient of 0.976. Specifically, the intrinsic motivation section achieved a Cronbach α of 0.967, the attitudes section 0.916, and the practices section 0.937, indicating excellent reliability across all dimensions.

The finalized questionnaire, written in Chinese (a version translated into English is present in Multimedia Appendix 1), included 5 dimensions with a total of 58 items. The basic information section comprised 16 items, while the intrinsic motivation section contained 9 subscales with 27 items. The attitudes dimension included 8 items, and the practices dimension consisted of 7 items. Scoring was based on the selection options and the number of items in each section, using a 5-point Likert scale for the intrinsic motivation, attitudes, and practices dimensions. Response options ranged from 5=strongly positive to 1=strongly negative. The scoring ranges were as follows: intrinsic motivation (27-135), attitudes (8-40), and practices (7-35). For intrinsic motivation, average scores of 27-67 were classified as low, 67-94 as moderate, and 95-135 as high. Attitude scores were categorized as negative (8-20), moderate (21-28), and positive (29-40). For practices, scores of 7-17 indicated negative practices, 18-24 moderate practices, and 25-35 positive practices. Participants scoring above 80% of the total were classified as positive performers [12]. This 80% threshold is a recognized benchmark in health care assessment studies to indicate adequate competency and positive performance [13].

### Data Collection and Quality Control of the Questionnaire

Participants from 12 hospitals in the eastern provinces of China (Shandong, Jiangsu, Shanghai, Zhejiang, and Fujian) were recruited by the WeChat (Tencent Holdings Ltd) groups using convenience sampling. We acknowledge that convenience sampling via WeChat groups may introduce selection bias. To mitigate this limitation, we recruited participants from various departments and specialties across these hospitals and encouraged department heads to distribute the survey link to their staff members, helping to reach physicians with varying levels of digital engagement. A web-based questionnaire and its QR code were created via the SoJump platform. The questionnaire link was distributed to participants via QR code or through the WeChat groups. Before answering the questions, participants were required to click the option “I agree to participate in this study” at the beginning of the e-questionnaire. All data were collected anonymously, and to prevent duplication, IP restriction was applied, allowing only 1 completion of the survey from a single IP address.

In order to further verify the reliability of the questionnaires, the Cronbach α for all valid questionnaires was 0.9801, and the Cronbach α values for the knowledge, attitudes, and practices sections were 0.9722, 0.9381, and 0.9822, respectively, demonstrating strong internal consistency across the total scale and its subscales. Additionally, the Kaiser-Meyer-Olkin value for the overall scale was 0.9306, suggesting the data were highly suitable for factor analysis.

### Sample Size

The sample size was calculated using the formula for cross-sectional studies: α=.05, 
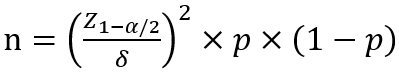
 where Z_1–α/2_=1.96 when α=.05, the assumed degree of variability of p=0.5 maximizes the required sample size, and δ is an admissible error (which was 5% here). The theoretical sample size was 480 which includes an extra 20% to allow for participants lost during the study.

### Statistical Analysis

Data analysis was conducted using SPSS (version 22.0; IBM Corp). Descriptive analysis was performed to summarize participants’ demographic characteristics and scores for intrinsic motivation, attitude, and practice. All continuous variables, including intrinsic motivation, attitude, and practice scores, were expressed as mean (SD), while categorical data, including demographic characteristics and individual question responses, were presented as n (%). For comparisons of intrinsic motivation, attitudes, and practices across demographic groups, continuous variables were first tested for normality. Normally distributed data were expressed as means (SD) and analyzed using independent sample 2-tailed *t* tests for 2-group comparisons. For nonnormally distributed data, the median (IQR) was reported and comparisons were conducted using the Mann-Whitney *U* test for 2 groups. For comparisons involving three or more groups, one-way ANOVA was applied to normally distributed data with equal variance, whereas the Kruskal-Wallis test was used for nonnormally distributed data. Pearson correlation analysis was used to assess relationships among intrinsic motivation, attitudes, and practices. The top 80% of scores for intrinsic motivation, attitude, and practice were used as cutoff values. Multivariate logistic regression analysis was performed to explore the association between demographic information and KAP scores. Variables in univariate analysis with a *P* value of less than .05 were included in multivariate analysis. Additionally, path analysis, a component of structural equation modeling (SEM), was used to explore relationships and potential mediation effects among intrinsic motivation, attitudes, practices, and demographic factors. A 2-sided *P* value of less than .05 was considered statistically significant for all statistical tests.

## Results

### Demographic Information

A total of 541 samples were initially collected. Following exclusions of (1) 1 case without “informed consent” and (2) 8 cases with response times under 120 seconds, the final dataset comprised 532 valid responses. Among these, 271 participants (50.9%) were female, 236 (44.4%) were aged 35-39 years, 275 (51.7%) held a bachelor’s degree, 437 (82.1%) worked in public tertiary hospitals, and 462 (86.8%) were employed in teaching hospitals. Additionally, 317 (59.6%) participants had not led or deeply participated in a research project, 249 (46.8%) reported being in a team with a strong research atmosphere, and 172 (32.3%) considered their mentors or discipline heads to have excellent research competence. The mean (SD) scores for intrinsic motivation, attitude, and practice were 108.79 (11.91), 32.23 (4.27), and 27.44 (3.81), respectively.

Analysis of demographic differences revealed that participants working in public tertiary hospitals were more likely to have lower attitude scores (*P*=.04). Moreover, participants who considered their mentors or discipline heads as having excellent research competence were more likely to exhibit higher intrinsic motivation, attitude, and practice scores (*P*=.03, *P*=.04, and *P*=.003, respectively; Table 1 and Table S1 in Multimedia Appendix 1).

**Table 1:** Demographic characteristics (N=532).

		Participants, n (%)	Intrinsic motivation			Attitude			Practice	
			Mean (SD)	*P* value	Mean (SD)		*P* value	Mean (SD)		*P* value
Total score		532 (100)	108.79 (11.91)		32.23 (4.27)			27.44 (3.81)		
**Sex**				.31			.06			.44
	Male	261 (49.1)	108.97 (13.39)		32.43 (4.72)			27.43 (4.21)		
	Female	271 (50.9)	108.62 (10.31)		32.03 (3.77)			27.45 (3.38)		
**Age (years)**				.87			.90			.81
	<35	160 (30.1)	109.08 (12.82)		32.39 (4.41)			27.56 (3.99)		
	35-39	236 (44.4)	109.18 (9.00)		32.28 (3.62)			27.33 (3.53)		
	≥40	136 (25.6)	107.79 (14.91)		31.94 (5.08)			27.49 (4.06)		
**Number of research project led or deeply participated in**				.72			.71			.66
	None	317 (59.6)	108.41 (12.68)		32.11 (4.20)			27.32 (3.97)		
	1~2 projects	165 (31)	109.19 (11.15)		32.44 (4.29)			27.50 (3.79)		
	3-5 projects	50 (9.4)	109.86 (9.07)		32.26 (4.64)			28.02 (2.62)		
**Research atmosphere in your team**				.09			.94			.23
	Poor	72 (13.5)	107.01 (17.34)		31.64 (5.99)			26.68 (4.86)		
	Average	211 (39.7)	107.88 (10.19)		32.35 (3.77)			27.35 (3.59)		
	Good	249 (46.8)	110.08 (11.25)		32.29 (4.07)			27.73 (3.62)		
**Rate your mentor or department head’s research competence**				.03			.04			.003
	Poor or very poor	22 (4.2)	106.45 (14.44)		31.86 (6.18)			27.45 (5.92)		
	Average	148 (27.8)	105.95 (14.16)		31.44 (4.49)			26.61 (4.13)		
	Good	190 (35.7)	108.59 (10.28)		32.24 (4.04)			27.33 (3.21)		
	Excellent	172 (32.3)	111.75 (10.44)		32.94 (3.94)			28.27 (3.65)		

### Distribution of Responses to Intrinsic Motivation, Attitude, and Practice

For the intrinsic motivation dimension, 70 (13.2%) of participants disagreed and 32 (6%) strongly disagreed that research work alleviates the monotony and fatigue of clinical work (I24). Additionally, 50 (9.4%) disagreed and 22 (4.1%) strongly disagreed that they could balance work and personal life while conducting research (I22), and 43 (8.1%) disagreed and 19 (3.6%) strongly disagreed about having opportunities to participate in or lead various research projects to gain practical experience (I27; Table S2 in Multimedia Appendix 1).

In the attitude dimension, 21 (3.9%) disagreed and 15 (2.8%) strongly disagreed that scientific research significantly improves clinical diagnosis and treatment standards (A1). Similarly, 25 (4.7%) disagreed and 16 (3%) strongly disagreed that improving research skills enhances the professional level of clinical physicians (A7). However, 136 (25.6%) strongly agreed, and 190 (35.7%) agreed that their hospital invests insufficiently in scientific research (A8; Table S3 in Multimedia Appendix 1).

Regarding the practice dimension, 30 (5.6%) disagreed and 16 (3%) strongly disagreed with discussing new research findings with colleagues to explore clinical applications (P7). Furthermore, 24 (4.5%) disagreed and 17 (3.2%) strongly disagreed with participating in research seminars, workshops, or events to gain insights and apply them clinically (P6), while 22 (4.1%) disagreed and 12 (2.3%) strongly disagreed with keeping track of the latest research developments and attempting to apply them in clinical practice (P4; Table S4 in Multimedia Appendix 1).

### Correlation Analysis

Correlation analysis showed significant positive associations between intrinsic motivation and attitude (*r*=0.444; *P*<.001) as well as between intrinsic motivation and practice (*r*=0.371; *P*<.001). A correlation was also observed between attitude and practice (*r*=0.354, *P*<.001; Table S5 in Multimedia Appendix 1).

### Univariate and Multivariate Analyses

The top 80% of scores for intrinsic motivation, attitude, and practice were used as cutoff values, resulting in 327 (61.5%) participants above the cutoff for intrinsic motivation, 315 (59.2%) for attitude, and 294 (55.3%) for practice (Table S6 in Multimedia Appendix 1). Multivariate logistic regression showed that no factors were independently associated with intrinsic motivation (Table 2). Meanwhile, the intrinsic motivation score (odds ratio [OR] 1.082, 95% CI 1.057-1.108; *P*<.001) was independently associated with a positive attitude (Table 3). Furthermore, intrinsic motivation score (OR 1.063, 95% CI 1.035-1.091, *P*<.001), attitude score (OR 1.095, 95% CI 1.029-1.165; *P*=.004), and good research atmosphere (OR 1.915; 95% CI 1.038-3.533; *P*=.04) were independently associated with proactive practice (Table 4).

**Table 2:** Univariate and multivariate analyses for intrinsic motivation dimension.

		Univariate analysis						Multivariate analysis		
		OR^a^ (95% CI)			*P* value		OR (95% CI)			*P* value
**Sex**										
	Male	Ref^b^			—^c^		—			—
	Female	0.869 (0.618-1.222)			.42		—			—
**Age (years)**										
	<35				—		—			—
	35-39	1.269 (0.849-1.900)			.25		—			—
	≥40	1.061 (0.671-1.677)			.80		—			—
**Ethnicity**										
	Han	Ref			—		—			—
	Ethnic minority	0.661 (0.129-3.028)			.59		—			—
**Marital status**										
	Unmarried	Ref			—		—			—
	Married	0.881 (0.583-1.325)			.54		—			—
**Given birth (number of children)**										
	No				—		—			—
	Yes	1.056 (0.708-1.575)			.79		—			—
**Mortgage or debt pressure**										
	No	Ref			—		—			—
	Yes	1.026 (0.705-1.490)			.89		—			—
**Field of specialization**										
	Internal medicine	Ref			—		—			—
	Surgery	1.162 (0.582-2.327)			.67		—			—
	Anesthesiology	1.106 (0.614-1.993)			.74		—			—
	Emergency medicine	1.140 (0.591-2.200)			.69		—			—
	Other	1.462 (0.607-3.592)			.40		—			—
**Education**										
	Bachelor’s degree	Ref			—		—			—
	Master’s degree	0.854 (0.596-1.223)			.39		—			—
	Doctorate or above	1.202 (0.641-2.291)			.57		—			—
**Hospital level**										
	Public tertiary hospital		Ref	—		—			—	
	Other		1.207 (0.774-1.896)	.41		—			—	
**Teaching hospital**										
	No	Ref			—		—			—
	Yes	1.149 (0.694-1.903)			.59		—			—
**Average monthly income per capita income (US $)**										
	5000 or less	Ref			—		—			—
	5001-10,000	1.524 (0.716-3.295)			.27		—			—
	10,001-20,000	1.280 (0.598-2.781)			.53		—			—
	More than 20,000	1.619 (0.581-4.611)			.36		—			—
	Prefer not to disclose	1.284 (0.552-3.021)			.56		—			—
**Relatives working in research**										
	No	Ref								
	Yes	1.051 (0.690-1.604)			.82		—			—
	Unsure	0.893 (0.301-2.653)			.84		—			—
**Number of research project led or deeply participated in**										
	None	Ref			—		Ref			—
	1~2 projects	1.080 (0.741-1.576)			.69		1.060 (0.725-1.551)			.76
	3-5 projects	1.542 (0.843-2.885)			.17		1.371 (0.734-2.562)			.32
**Number of research training or seminars you have attended**										
	None	Ref			—		Ref			—
	Approximately 1-2 times	1.015 (0.654-1.574)			.95		1.016 (0.654-1.578)			.95
	3-5 times	1.080 (0.597-1.958)			.80		1.074 (0.593-1.944)			.81
	More than 5 times	1.586 (0.989-2.556)			.057		1.527 (0.944-2.470)			.08
**Research atmosphere in your team**										
	Poor	Ref			—		—			—
	Average	0.778 (0.452-1.329)			.36		—			—
	Good	0.995 (0.584-1.684)			.98		—			—
**Rate your mentor or department head’s research competence**										
	Poor or very poor	Ref			—		—			—
	Average	0.897 (0.363-2.220)			.81		—			—
	Good	1.043 (0.427-2.548)			.93		—			—
	Excellent	1.529 (0.622-3.764)			.35		—			—

^a^OR: odds ratio.

^b^Reference values.

^c^Not applicable.

**Table 3:** Univariate and multivariate analyses for attitude dimension.

				Univariate analysis						Multivariate analysis		
				OR^a^ (95% CI)			*P* value		OR (95% CI)			*P* value
Intrinsic motivation				1.081 (1.057-1.106)			<.001		1.082 (1.057-1.108)			<.001
**Sex**												
	Male		Ref^b^			—^c^		Ref			—	
	Female		0.721 (0.509-1.020)			.07		0.801 (0.546-1.177)			0.26	
**Age (years)**												
	<35		Ref			—		—			—	
	35-39		1.080 (0.716-1.625)			.71		—			—	
	≥40		0.944 (0.594-1.502)			.81		—			—	
**Ethnicity**												
	Han		Ref			—		—			—	
	Ethnic minority		0.918 (0.200-4.697)			.91		—			—	
**Marital status**												
	Unmarried		Ref			—		Ref			—	
	Married				1.391 (0.922-2.098)		.12		1.239 (0.787-1.950)			.36
**Given birth (number of children)**												
		No	Ref			—		Ref			—	
		Yes	1.415 (0.946-2.114)			.09		1.325 (0.845-2.080)			.22	
**Mortgage or debt pressure**												
	No		Ref			—		—			—	
	Yes		0.940 (0.641-1.374)			.75		—			—	
**Field of specialization**												
	Internal medicine		Ref			—		—			—	
	Surgery		0.930 (0.453-1.891)			.84		—			—	
	Anesthesiology		0.692 (0.373-1.256)			.23		—			—	
	Emergency medicine		1.113 (0.559-2.191)			.76		—			—	
	Other		1.294 (0.517-3.362)			.59		—			—	
**Education**												
	Bachelor’s degree		Ref			—		—			—	
	Master’s degree		1.034 (0.718-1.491)			.89		—			—	
	Doctorate or above		0.991 (0.528-1.891)			.98		—			—	
**Hospital level**												
	Public tertiary hospital		Ref			—		—			—	
	Other		1.445 (0.913-2.325)			.12		1.341 (0.797-2.256)			.27	
**Teaching hospital**												
	No		Ref			—		—			—	
	Yes		0.781 (0.458-1.307)			.36		—			—	
**Average monthly income per capita income (US $)**												
	5000 or less		Ref			—		Ref			—	
	5001-10,000		2.265 (1.059-4.917)			.04		2.099 (0.919-4.796)			.08	
	10,001-20,000		1.691 (0.789,3.679)			.18		1.521 (0.663-3.490)			.32	
	More than 20,000		1.877 (0.671,5.406)			.23		1.609 (0.519-4.990)			.41	
	Prefer not to disclose		1.214 (0.522,2.856)			.65		1.205 (0.482-3.012)			.69	
**Relatives working in research**												
	No		Ref			—		Ref			—	
	Yes		0.859 (0.563-1.318)			.48		0.744 (0.469,1.179)			.21	
	Unsure		0.491 (0.159-1.437)			.20		0.370 (0.114,1.204)			.10	
**Number of research project led or deeply participated in**												
	None		Ref			—		—			—	
	1~2 projects		0.966 (0.660-1.419)			.86		—			—	
	3-5 projects		0.935 (0.513-1.730)			.83		—			—	
**Number of research training or seminars you have attended**												
	None		Ref			—		Ref			—	
	Approximately 1-2 times		0.949 (0.604-1.488)			.82		0.921 (0.559-1.519)			.75	
	3-5 times		0.656 (0.360-1.194)			.17		0.589 (0.306-1.132)			.11	
	More than 5 times		0.857 (0.530-1.382)			.53		0.683 (0.403-1.159)			.16	
**Research atmosphere in your team**												
	Poor		Ref			—		—			—	
	Average		1.194 (0.688-2.054)			.53		—			—	
	Good		0.933 (0.545-1.582)			.80		—			—	
**Rate your mentor or department head’s research competence**												
	Poor or very poor		Ref			—		—			—	
	Average		0.711 (0.278-1.750)			.46		—			—	
	Good		1.187 (0.468-2.893)			.71		—			—	
	Excellent		1.140 (0.448-2.791)			.78		—			—	

^a^OR: odds ratio.

^b^Reference values.

^c^Not applicable.

**Table 4:** Univariate and multivariate analysis for practice dimension.

				Univariate analysis				Multivariate analysis		
				OR^a^ (95% CI)		*P* value		OR (95% CI)		*P* value
Intrinsic motivation				1.082 (1.058-1.107)		<.001		1.063 (1.035-1.091)		<.001
Attitude				1.182 (1.124-1.243)		<.001		1.095 (1.029-1.165)		.004
**Sex**										
	Male		Ref^b^		—^c^		—		—	
	Female		0.919 (0.653-1.294)		.63		—		—	
**Age (years)**										
	Under 35		Ref		—		—		—	
	35-39		1.101 (0.735-1.648)		.64		—		—	
	More than 40		1.187 (0.749-1.883)		.47		—		—	
**Ethnicity**										
	Han		Ref		—		—		—	
	Ethnic minority		1.081 (0.236-5.530)		.92		—		—	
**Marital status**										
	Unmarried		Ref		—		—		—	
	Married		0.906 (0.599-1.366)		.64		—		—	
**Given birth (number of children)**										
		No	Ref		—		—		—	
		Yes	1.142 (0.765-1.703)		.52		—		—	
**Mortgage or debt pressure**										
	No		Ref		—		—		—	
	Yes		0.903 (0.618,1.314)		.59		—		—	
**Field of specialization**										
	Internal medicine		Ref		—		Ref		—	
	Surgery		0.778 (0.382-1.565)		.48		0.692 (0.312-1.531)		.36	
	Anesthesiology		0.632 (0.343-1.142)		.13		0.602 (0.306-1.182)		.14	
	Emergency medicine		1.156 (0.584-2.265)		.68		1.103 (0.520-2.338)		.80	
	Other		0.930 (0.381-2.295)		.87		0.805 (0.303-2.140)		.66	
**Education**										
	Bachelor’s degree		Ref		—		Ref		—	
	Master’s degree		0.898 (0.627-1.288)		.56		0.978 (0.654-1.462)		.91	
	Doctorate or above		1.672 (0.877-3.316)		.13		1.813 (0.870-3.778)		.11	
**Hospital level**										
	Public tertiary hospital		Ref		—		—		—	
	Other		1.081 (0.692-1.698)		.73		—		—	
**Teaching hospital**										
	No		Ref		—		—		—	
	Yes		0.856 (0.511-1.420)		.55		—		—	
**Average monthly income per capita income (US $)**										
	5000 or less		Ref		—		Ref		—	
	5001-10,000		1.841 (0.863-4.025)		.12		1.785 (0.768-4.147)		.18	
	10,001-20,000		1.694 (0.790-3.723)		.18		1.606 (0.691-3.730)		.27	
	More than 20,000		2.140 (0.764-6.206)		.15		2.253 (0.720-7.050)		.16	
	Prefer not to disclose		1.636 (0.703-3.892)		.26		1.919 (0.752-4.896)		.17	
**Relatives working in research**										
	No		Ref		—		Ref		—	
	Yes		1.210 (0.792-1.861)		.38		1.118 (0.702-1.782)		.64	
	Unsure		0.458 (0.139-1.351)		.17		0.457 (0.130-1.600)		.22	
**Number of research project led or deeply participated in**										
	None		Ref		—		—		—	
	1~2 projects		1.249 (0.855-1.832)		.25		—		—	
	3-5 projects		1.115 (0.613-2.049)		.72		—		—	
**Number of research training or seminars you have attended**										
	None		Ref		—		—		—	
	Approximately 1-2 times		0.856 (0.549-1.331)		.49		—		—	
	3-5 times		0.890 (0.491-1.621)		.70		—		—	
	More than 5 times		0.913 (0.569-1.465)		.71		—		—	
**Research atmosphere in your team**										
	Poor		Ref		—		Ref		—	
	Average		1.067 (0.625-1.826)		.81		1.202 (0.645-2.237)		.56	
	Good		1.685 (0.994-2.864)		.053		1.915 (1.038-3.533)		.04	
**Rate your mentor or department head’s research competence**										
	Poor or very poor		Ref		—		—		—	
	Average		0.499 (0.189-1.237)		.14		0.434 (0.149-1.259)		.12	
	Good		0.691 (0.265-1.692)		.43		0.466 (0.162-1.342)		.16	
	Excellent		0.941 (0.359-2.321)		.90		0.557 (0.193-1.611)		.28	

^a^OR: odds ratio.

^b^Reference values.

^c^Not applicable.

### SEM Analysis

**Figure 1:**
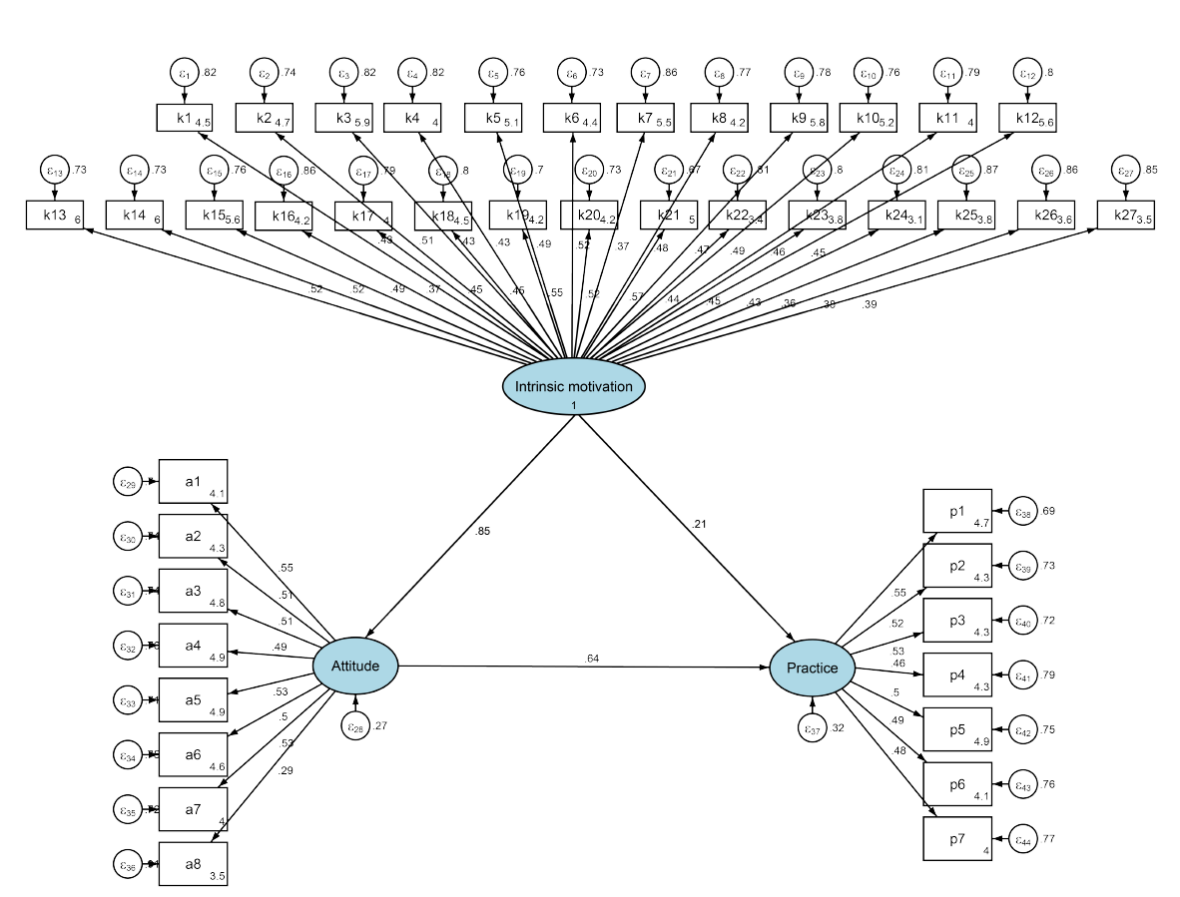
SEM model. The figure was created by the AMOS software (version 26.0; IBM Corp). AMOS: Analysis of Moment Structures; SEM: structural equation model. Intrinsic motivation had a direct positive effect on attitude, attitude directly affected practice.

The SEM showed an excellent fit (root-mean-square error of approximation=0.027; standardized root-mean-square residual=0.039; Tucker-Lewis index=0.921; comparative fit index=0.925; Table S7 in Multimedia Appendix 1). Specific relationships between intrinsic motivation, attitude, and practice are detailed in Table S8 in Multimedia Appendix 1 and Figure 1. Mediation analysis based on the SEM revealed that intrinsic motivation had a direct positive effect on attitude (β=0.854; *P*<.001), attitude directly affected practice (β=0.637; *P*<.001), and intrinsic motivation indirectly influenced practice through attitude (β=0.544; *P*<.001; Table S9 in Multimedia Appendix 1).

The structural equation model is a comprehensive statistical approach to testing hypotheses about relations among latent (intrinsic motivation, attitude, and practice) and observed variables (the questions for each dimension). (1) Graphic shape: The latent variables are typically represented by ellipses or circles, observed variables are generally depicted as rectangles or squares, and error terms are illustrated by small circles connected to the corresponding observed variables. (2) Arrows: The arrows and coefficients among the three dimensions constructs indicate both the direction and strength of the relationships between the latent variables. The small circles associated with A and P represent the residual terms. A unidirectional arrow signifies the direction of causation, specifically from cause to effect. When an arrow points from one latent variable to another, it indicates that the former exerts a direct effect on the latter. Conversely, if the latent variable is directed from the observed variable, it signifies that the latent variable is represented by that observed variable. (3) Number: The numerical value on the arrow connecting the latent variable to the observed variable signifies the factor loading, which reflects the strength of the association between the observed variable and the latent variable, specifically the regression coefficient of the observed variable with respect to the latent variable. A positive value indicates a positive correlation, suggesting that as the latent variable increases, the observed variable also tends to increase; conversely, a negative value indicates a negative correlation, implying that as the latent variable increases, the observed variable tends to decrease.

## Discussion

The main findings of this study revealed 3 key insights. First, young physicians demonstrated high levels of intrinsic motivation (mean 108.79, SD 11.91) and positive attitudes (mean 32.23, SD 4.27) toward scientific research, but exhibited relatively less active research practices (mean 27.44, SD 3.81). Second, intrinsic motivation directly influenced attitudes and indirectly affected practices through attitudes as confirmed by SEM. Third, a supportive research atmosphere within teams emerged as an independent factor associated with proactive research practices (OR 1.915, 95% CI 1.038-3.533). These findings highlight a gap between psychological readiness and actual engagement in research among young physicians in China. Targeted interventions aimed at fostering a supportive research environment and enhancing practical engagement in research activities could help translate motivation and attitudes into proactive research practices among young physicians.

The discrepancy between motivation and practice is consistent with findings in previous research, which identified similar gaps among health care professionals across various settings [14,15]. While young physicians recognize the value of research in advancing clinical knowledge and professional growth, systemic and environmental barriers may limit their ability to engage actively in research. This underperformance in the practice dimension may contribute to slower adoption of evidence-based medicine, a challenge previously reported in studies investigating research involvement among physicians [16,17]. It may also be a contributing factor to suboptimal patient care and limited progress in clinical innovations.

In terms of the relationships between intrinsic motivation, attitudes, and practices, the results from correlation analyses and SEM provide important insights with notable effect sizes. The correlation analysis revealed moderate positive associations between intrinsic motivation and attitude (*r*=0.444; *P*<.001), intrinsic motivation and practice (*r*=0.371; *P*<.001), and between attitude and practice (*r*=0.354; *P*<.001), indicating meaningful relationships between these constructs. More importantly, the SEM demonstrated that intrinsic motivation had a strong direct effect on attitudes (β=0.854; *P*<.001, explaining approximately 73% of the variance in attitudes) and a significant indirect effect on practices through attitudes (β=0.544; *P*<.001). Additionally, attitudes directly affected practices (β=0.637; *P*<.001, explaining approximately 41% of the variance in practices). These standardized coefficients indicate substantial relationships, suggesting that enhancing intrinsic motivation could significantly improve attitudes toward research, which in turn would promote more active research practices. These findings align with other studies that emphasize the role of intrinsic motivation as a foundational driver of engagement in scientific research [18,19]. Intrinsic motivation fosters a sense of curiosity, problem-solving, and personal fulfillment, which are often associated with positive attitudes toward research. However, the indirect nature of its effect on practices underscores the presence of intervening barriers—such as workload, insufficient institutional support, and inadequate mentorship—that hinder the translation of motivation into action [20,21].

The relatively inactive practices reported by the participants in this study are consistent with findings from other research that highlight a lack of resources, limited access to research opportunities, and time constraints as common obstacles [22,23]. Our findings reveal specific systemic and environmental barriers that hinder young physicians’ research engagement, which merit more detailed examination. First, workload pressures emerged as a significant barrier, with 19.4% of participants disagreeing that research alleviates clinical monotony (item I24) and 13.5% reporting difficulty balancing research with personal life (item I22). This aligns with our finding that intrinsic motivation alone does not directly translate to practice without supportive environmental factors. Second, inadequate institutional support was explicitly identified, with 61.3% of respondents agreeing that their hospitals invest insufficiently in scientific research. This is particularly concerning as our multivariate analysis demonstrated that a good research atmosphere significantly predicted active research practices (OR 1.915, 95% CI 1.038-3.533). The variation in practice scores based on the perceived research competence of mentors further suggests that inadequate mentorship and role modeling represent significant environmental barriers. Third, our data revealed uneven access to research opportunities, with 11.7% of participants disagreeing that they had opportunities to participate in or lead research projects (item I27). This access gap creates a structural barrier that prevents intrinsically motivated physicians from gaining the practical experience necessary to develop research competence. For instance, a study conducted among early-career physicians in other countries identified similar challenges, with time pressures from clinical duties being the most cited reason for low research engagement [24,25]. Moreover, the positive attitudes observed here mirror findings in other studies, which suggest that young health care professionals are often optimistic about the value of research in improving health care outcomes and advancing their careers [26,27].

Conversely, this study’s finding that intrinsic motivation is universally high across demographic groups contrasts with some previous research, which identified variations in motivation based on factors such as educational background, sex, and age [28,29]. This suggests that while motivation is a strong starting point, addressing structural barriers may have a more significant impact on improving research practices.

The observed significant differences in practice scores based on team research atmosphere and the perceived competence of mentors or department heads highlight the importance of the surrounding environment. Participants in teams with strong research atmospheres and those with highly competent mentors were more likely to report better research practices. This observation aligns with studies that emphasize the role of mentorship and collaborative team dynamics in fostering research engagement [30,31]. Effective mentors can provide guidance, inspire confidence, and facilitate access to resources, while a positive research atmosphere encourages peer collaboration and reduces feelings of isolation in research efforts. Notably, demographic variables (sex, age, and education) showed no associations with research outcomes, unlike some international studies [32,33]. This may reflect sample homogeneity, gender equity progress in Chinese medical education, standardized training systems, and cultural factors emphasizing hierarchical structures. These findings suggest interventions should prioritize environmental factors over targeting demographic groups. Interestingly, other variables such as gender, age, and educational background did not demonstrate significant associations with practices. This suggests that these factors may not be critical determinants of research engagement in this population. Instead, institutional and environmental factors appear to play a more prominent role. For example, despite intrinsic motivation being high across all participants, its effect on practice is significantly moderated by external factors such as team support and resource availability.

To address the deficiencies observed in practice, targeted, evidence-based interventions are needed. First, institutions should consider implementing dedicated research time for young physicians. For example, protected research time during clinical rotations has been shown to improve research output in similar contexts [34,35]. Additionally, reducing non–research-related administrative burdens may help physicians dedicate more time to scholarly activities. Second, mentorship programs should be expanded and formalized. Institutions can pair young physicians with experienced mentors, provide regular feedback sessions, and offer structured research training programs. Evidence from previous studies suggests that such programs significantly enhance both research skills and confidence among early-career health care professionals [36,37]. It is important to acknowledge the implementation challenges these interventions may face in China’s health care context. Protected research time, while effective, may be difficult to implement in hospitals with high patient volumes and physician shortages. Mentorship programs, though relatively cost-effective, require investment in mentor training and coordination. Financial incentives present budgetary challenges, especially for nontertiary hospitals, making promotion-linked research recognition a more feasible alternative. Infrastructure development represents the most resource-intensive intervention and may require tiered implementation or resource-sharing models across hospital networks to be realistically achievable in varied settings.

Third, tangible incentives should be provided to encourage research engagement. These could include financial support for research projects, conference attendance sponsorships, or recognition for research achievements through awards and promotions. Additionally, hospitals should establish clear pathways linking research contributions to career advancement opportunities, such as promotions or leadership roles, to motivate young physicians. Fourth, addressing infrastructure gaps is critical. Institutions must ensure access to adequate research resources, including funding, technical support, and state-of-the-art equipment. The findings from this study align with those of previous research, which showed that lack of resources significantly hinders research engagement [38,39]. Policy makers and hospital administrators should prioritize investments in research infrastructure to foster a culture of inquiry and innovation. Fifth, targeted interventions for specific groups of young physicians, such as those in surgery or anesthesiology who reported relatively lower practice scores, should be considered. For instance, integrating research requirements into residency programs or creating specialty-specific research opportunities may encourage greater involvement. Finally, efforts to improve the research atmosphere within teams should include fostering collaboration through regular research meetings, multidisciplinary discussions, and team-building activities. Institutions could also introduce peer mentorship programs to facilitate knowledge exchange and strengthen team cohesion [40,41].

Several aspects of intrinsic motivation, attitudes, and practices require particular attention. For example, a significant proportion of participants reported neutral or negative responses regarding their ability to balance work and personal life while conducting research, as well as their access to research facilities and technical support. These challenges have also been highlighted in other studies as barriers to research engagement [42,43]. Addressing these issues requires specific measures, such as providing flexible work schedules, improving access to shared research facilities, and offering technical training workshops. Similarly, many participants reported insufficient support from their institutions in creating a conducive research environment. Hospitals should create policies to actively support young researchers by offering financial aid, creating junior investigator grants, and establishing dedicated research offices to assist with project management. Additionally, fostering a positive research culture through leadership initiatives and institutional support is critical for motivating physicians to engage actively in research [44,45].

This study has several limitations. First, as a cross-sectional survey, it only provides a snapshot of young physicians' intrinsic motivation, attitudes, and practices, making it difficult to infer causal relationships. While our SEM analysis suggests directional relationships between intrinsic motivation, attitudes, and practices, the temporal sequence and true causality cannot be definitively established. Future longitudinal or intervention studies are needed to validate these proposed relationships and confirm the directionality of these associations. Second, the data rely on self-reported questionnaires, which may be subject to response bias, including social desirability bias. Third, our recruitment strategy using WeChat groups may have introduced selection bias by potentially overrepresenting physicians who are more digitally engaged or active in professional web-based networks. However, the widespread use of WeChat for professional communication among Chinese health care workers minimizes this concern. Finally, although this study involved multiple centers in eastern China, many regions across the country were not included, which may limit the generalizability of the findings to other areas or diverse health care settings. Importantly, our sample predominantly consisted of physicians from urban teaching hospitals in economically developed eastern provinces, which may not represent the experiences of physicians in rural areas, western regions, or nonteaching hospitals where research resources, infrastructure, and priorities may differ significantly.

In conclusion, young physicians demonstrated positive intrinsic motivation and attitudes but relatively inactive practices toward scientific research and its clinical value, with intrinsic motivation significantly influencing attitudes and practices both directly and indirectly. To enhance scientific research engagement among young physicians, targeted interventions should focus on fostering a supportive research atmosphere and providing practical opportunities to translate motivation and attitudes into proactive research practices.

## Appendix

Multimedia Appendix 1 Additional material.

## Abbreviations

KAP: Knowledge, Attitude, and Practice
OR: odds ratio
SEM: structural equation modeling

## Data Availability

All data generated or analyzed during this study are included in this published article.
