# Supplementary material for: Intrinsic Motivation, Attitudes, and Practices of Young Physicians Regarding Scientific Research: Cross-Sectional Study in China
Source: J Med Internet Res. 2025 Apr 22;27:e72633. doi: 10.2196/72633 (PMC12056413; doi:10.2196/72633)
Supplement: Multimedia Appendix 1 [file jmir_v27i1e72633_app1.docx]

**Supplement materials**

**Table S1 Distribution of Intrinsic Motivation Dimension Responses**

| **Knowledge** | Strongly agree | Agree | Neutral | Disagree | Strongly disagree |
| --- | --- | --- | --- | --- | --- |
| 1. Scientific research helps me gain a deeper understanding of medical knowledge and disease mechanisms. | 204(38.3%) | 197(37%) | 108(20.3%) | 17(3.2%) | 6(1.1%) |
| 2. Participating in research enhances my clinical skills and innovation abilities. | 204(38.3%) | 203(38.2%) | 111(20.9%) | 7(1.3%) | 7(1.3%) |
| 3. Research experience helps me build higher prestige in my professional field. | 245(46.1%) | 216(40.6%) | 65(12.2%) | 5(0.9%) | 1(0.2%) |
| 4. I have a strong interest in scientific research and enjoy the problem-solving process. | 159(29.9%) | 196(36.8%) | 141(26.5%) | 24(4.5%) | 12(2.3%) |
| 5. Successfully completing research projects and publishing in SCI journals gives me a great sense of accomplishment. | 210(39.5%) | 222(41.7%) | 85(16%) | 11(2.1%) | 4(0.8%) |
| 6. Engaging in research makes me feel fulfilled and represents an important aspect of my personal value. | 205(38.5%) | 191(35.9%) | 111(20.9%) | 15(2.8%) | 10(1.9%) |
| 7. Research achievements are essential for my career advancement. | 280(52.6%) | 191(35.9%) | 48(9%) | 7(1.3%) | 6(1.1%) |
| 8. I believe research skills are an indispensable core competency for modern physicians. | 203(38.2%) | 192(36.1%) | 108(20.3%) | 14(2.6%) | 15(2.8%) |
| 9. Through research, I can gain more career development opportunities (e.g., advanced education, overseas training, participation in high-level seminars). | 208(39.1%) | 255(47.9%) | 62(11.7%) | 3(0.6%) | 4(0.8%) |
| 10. I hope to make a positive impact on the medical field through my research findings. | 207(38.9%) | 247(46.4%) | 62(11.7%) | 9(1.7%) | 7(1.3%) |
| 11. I believe participating in research is a direct contribution to society and patient health. | 218(41%) | 185(34.8%) | 88(16.5%) | 27(5.1%) | 14(2.6%) |
| 12. Publishing high-quality research papers can enhance my influence in the academic community at home and abroad. | 210(39.5%) | 234(44%) | 82(15.4%) | 4(0.8%) | 2(0.4%) |
| 13. Participating in research teams allows me to collaborate with top experts and broaden my horizons. | 206(38.7%) | 275(51.7%) | 42(7.9%) | 4(0.8%) | 5(0.9%) |
| 14. Teamwork in research helps me build an extensive network of contacts. | 219(41.2%) | 249(46.8%) | 58(10.9%) | 4(0.8%) | 2(0.4%) |
| 15. Through research collaboration, I can learn knowledge and skills from other fields. | 243(45.7%) | 234(44%) | 44(8.3%) | 4(0.8%) | 7(1.3%) |
| 16. The economic returns from research achievements (e.g., bonuses, patent income) are a motivation for me to engage in research. | 146(27.4%) | 227(42.7%) | 126(23.7%) | 21(3.9%) | 12(2.3%) |
| 17. Incentive policies for research achievements from the hospital or institution strongly motivate me. | 126(23.7%) | 225(42.3%) | 143(26.9%) | 20(3.8%) | 18(3.4%) |
| 18. Research project funding provides me with stable financial support, reducing the economic burden of conducting research. | 151(28.4%) | 230(43.2%) | 120(22.6%) | 29(5.5%) | 2(0.4%) |
| 19. I enjoy the challenges that research brings, as it pushes me to continuously exceed my limits. | 187(35.2%) | 197(37%) | 120(22.6%) | 16(3%) | 12(2.3%) |
| 20. Solving research problems ignites my passion for continuous learning and improvement. | 180(33.8%) | 241(45.3%) | 81(15.2%) | 8(1.5%) | 22(4.1%) |
| 21. Research work keeps me attuned to new knowledge and technologies. | 186(35%) | 241(45.3%) | 90(16.9%) | 8(1.5%) | 7(1.3%) |
| 22. I am able to balance work and personal life while conducting research. | 154(28.9%) | 173(32.5%) | 133(25%) | 50(9.4%) | 22(4.1%) |
| 23. My hospital or department provides sufficient support and understanding for young doctors involved in research, helping to reduce work pressure. | 158(29.7%) | 210(39.5%) | 111(20.9%) | 40(7.5%) | 13(2.4%) |
| 24. I believe that research work helps alleviate the monotony and fatigue of clinical work. | 125(23.5%) | 173(32.5%) | 132(24.8%) | 70(13.2%) | 32(6%) |
| 25. My hospital or department has a positive research atmosphere that encourages and supports young doctors in conducting research. | 148(27.8%) | 225(42.3%) | 112(21.1%) | 24(4.5%) | 23(4.3%) |
| 26. I have access to the necessary research facilities, equipment, and technical support. | 123(23.1%) | 195(36.7%) | 150(28.2%) | 47(8.8%) | 17(3.2%) |
| 27. I have opportunities to participate in or lead various research projects, providing ample practical experience. | 153(28.8%) | 178(33.5%) | 139(26.1%) | 43(8.1%) | 19(3.6%) |

**Table S2 Distribution of Attitude** **Dimension Responses**

| **Attitude** | Strongly agree | Agree | Neutral | Disagree | Strongly disagree |
| --- | --- | --- | --- | --- | --- |
| 1. I believe that scientific research significantly contributes to improving clinical diagnosis and treatment standards. | 172(32.3%) | 220(41.4%) | 104(19.5%) | 21(3.9%) | 15(2.8%) |
| 2. I believe that research findings can directly enhance strategies for disease prevention, diagnosis, and treatment. | 182(34.2%) | 221(41.5%) | 97(18.2%) | 21(3.9%) | 11(2.1%) |
| 3. I believe that research discoveries help uncover disease mechanisms, providing scientific foundations for clinical decision-making. | 199(37.4%) | 244(45.9%) | 66(12.4%) | 12(2.3%) | 11(2.1%) |
| 4. I believe that research findings facilitate the development of new drugs, medical devices, or treatment methods. | 202(38%) | 242(45.5%) | 66(12.4%) | 13(2.4%) | 9(1.7%) |
| 5. I believe that clinical physicians should actively follow and apply new research findings in clinical practice. | 201(37.8%) | 250(47%) | 62(11.7%) | 7(1.3%) | 12(2.3%) |
| 6. I believe that research progress increases my confidence in explaining treatment plans to patients. | 191(35.9%) | 250(47%) | 61(11.5%) | 17(3.2%) | 13(2.4%) |
| 7. I believe that improving research skills can enhance the professional level of clinical physicians. | 178(33.5%) | 217(40.8%) | 96(18%) | 25(4.7%) | 16(3%) |
| 8. I believe that my hospital does not invest enough in scientific research. | 136(25.6%) | 190(35.7%) | 130(24.4%) | 62(11.7%) | 14(2.6%) |

**Table S3 Distribution of Practice Dimension Responses**

| **Practice** | Strongly agree | Agree | Neutral | Disagree | Strongly disagree |
| --- | --- | --- | --- | --- | --- |
| 1. I actively participate in research activities that enhance my professional skills. | 136(25.6%) | 237(44.5%) | 144(27.1%) | 8(1.5%) | 7(1.3%) |
| 2. I incorporate new research findings into patient education to support informed decision-making. | 147(27.6%) | 230(43.2%) | 129(24.2%) | 15(2.8%) | 11(2.1%) |
| 3. I regularly review research literature to update my clinical knowledge. | 153(28.8%) | 234(44%) | 108(20.3%) | 29(5.5%) | 8(1.5%) |
| 4. I keep track of the latest research developments in my field and try to apply them in clinical practice. | 152(28.6%) | 239(44.9%) | 107(20.1%) | 22(4.1%) | 12(2.3%) |
| 5. When facing clinical issues, I proactively consult research literature to find solutions. | 149(28%) | 273(51.3%) | 89(16.7%) | 15(2.8%) | 6(1.1%) |
| 6. I actively participate in research seminars, workshops, and other events to gain insights and apply them clinically. | 141(26.5%) | 247(46.4%) | 103(19.4%) | 24(4.5%) | 17(3.2%) |
| 7. I discuss new research findings with colleagues to explore the clinical applications together. | 133(25%) | 241(45.3%) | 112(21.1%) | 30(5.6%) | 16(3%) |

**Table S4. Spearman Correlation Analysis**

| **Spearman** | **Intrinsic Motivation** | **Attitude** | **Practice** |
| --- | --- | --- | --- |
| **Intrinsic Motivation** | 1.000 |  |  |
| **Attitude** | 0.444 (P<0.001) | 1.000 |  |
| **Practice** | 0.371 (P<0.001) | 0.354 (P<0.001) | 1.000 |

**Table S5. Univariate and multivariate analysis**

| **Cutoff value：80% of the total score** | N(%) |
| --- | --- |
| **Intrinsic Motivation total score** |  |
| Ksum>=108 | 327(61.47%) |
| Ksum<=107 | 205(38.53%) |
| Attitude total score |  |
| Asum>=32 | 315(59.21%) |
| Asum<=31 | 217(40.79%) |
| Practice total score |  |
| Psum>=28 | 294(55.26%) |
| Psum<=27 | 238(44.74%) |

**Table S6. SEM model fit.**

| **Indicators** | **Reference** | **Results** |
| --- | --- | --- |
| RMSEA | <0.08 | 0.027 |
| SRMR | <0.08 | 0.039 |
| TLI | >0.80 | 0.921 |
| CFI | >0.80 | 0.925 |

**Table S7.** **Estimate of Total Effects for SEM.**

| **Indicators** |  | **Estimate** | **P>\|z\|** |
| --- | --- | --- | --- |
| Attitude |  |  |  |
|  | **Intrinsic motivation** | 34.08 | <0.001 |
| Practice |  |  |  |
|  | **Intrinsic motivation** | 23.89 | <0.001 |
|  | Attitude | 4.88 | <0.001 |

**Table S8. Mediation Analysis based on the SEM.**

| **Model paths** | | Total effects | | Direct effects | | Indirect effects | |
| --- | --- | --- | --- | --- | --- | --- | --- |
|  |  | β(95%CI) | P | β(95%CI) | P | β(95%CI) | P |
| Attitude |  |  |  |  |  |  |  |
|  | **Intrinsic motivation** | 0.854 (0.804, 0.903) | <0.001 | 0.854 (0.804, 0.903) | <0.001 |  |  |
| Practice |  |  |  |  |  |  |  |
|  | **Intrinsic motivation** | 0.758 (0.696, 0.820) | <0.001 | 0.214 (-0.038, 0.467) | 0.096 | 0.544 (0.314, 0.774) | <0.001 |
|  | Attitude | 0.637 (0.381, 0.893) | <0.001 | 0.637 (0.381, 0.893) | <0.001 |  |  |
